# Supplementary figures and images for: Chromatin maturation of the HIV-1 provirus in primary resting CD4+ T cells
Source: PLoS Pathog. 2020 Jan 30;16(1):e1008264. doi: 10.1371/journal.ppat.1008264 (PMC6991963; doi:10.1371/journal.ppat.1008264)

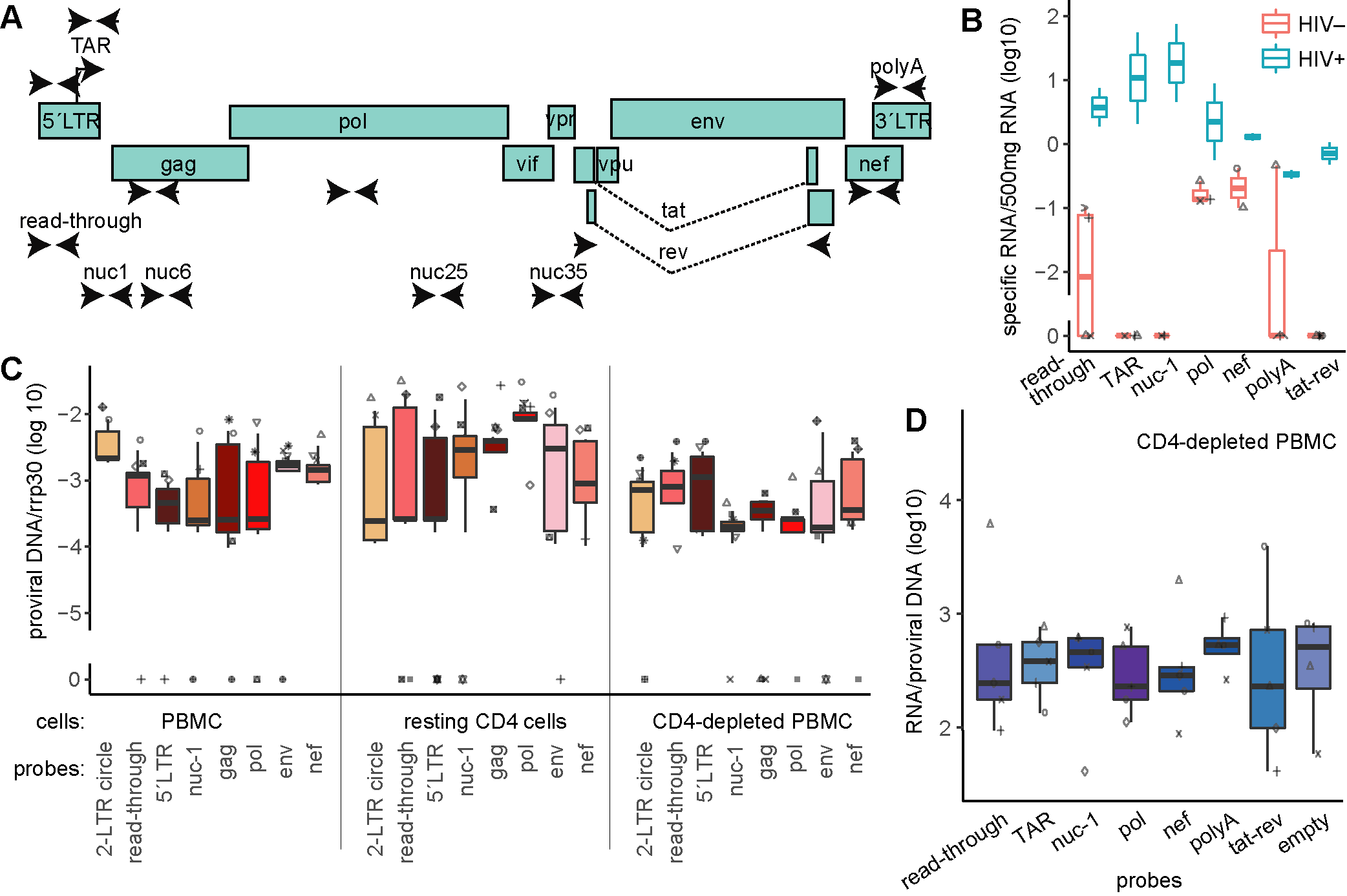

Supplement: S1 Fig — (A) Primer positions relative to the HIV-1 provirus. Viral proteins depicted with blue bars. (B) RT-ddPCR using primers for HIV-1 tested on RNA from CD4 cells isolated from HIV-1 negative (HIV–) (n = 4) and HIV-1 positive (HIV+) (in total n = 10, here represented by n = 2) study participants, values normalized to 500ng RNA. Axis is broken to depict that for all but the pol and nef primer probe combinations, no HIV-1 signal was consistently detected in cells from HIV-1 negative donors (C) ddPCR of DNA from the cells originating from HIV-1 positive study participants. Probe efficiency identified cells where the primer-probe combinations were able to detect genomic proviral DNA (n = 10). (D) RT-ddPCR on RNA isolated from CD4-depleted T-cells from HIV-1 positive study participants (n = 5). (TIF) [file ppat.1008264.s001.tif]

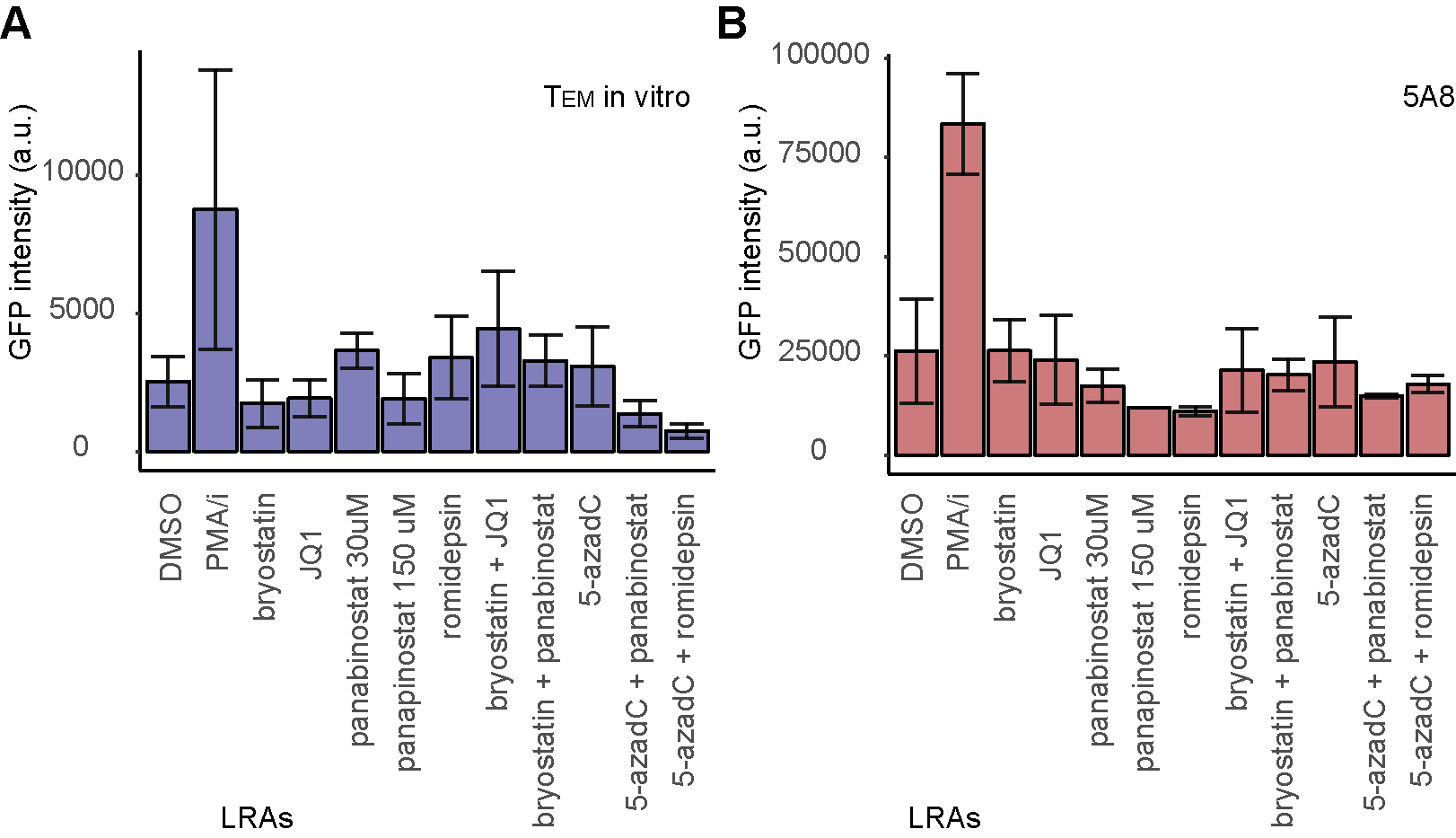

Supplement: S2 Fig — Cells were exposed to different agents for 24 hours, after which cells were fixed and the intensify of GFP among the GFP-positive cells were recorded by flow cytometry. 5-azadC in all conditions (alone or in combination) was added 72 hours prior to fixation. (A) In vitro HIV-1 infected TEM cells from HIV-1 negative study participants (n = 4) and (B) J-lat 5A8 cells (n = 2, technical replicates). (TIF) [file ppat.1008264.s002.tif]

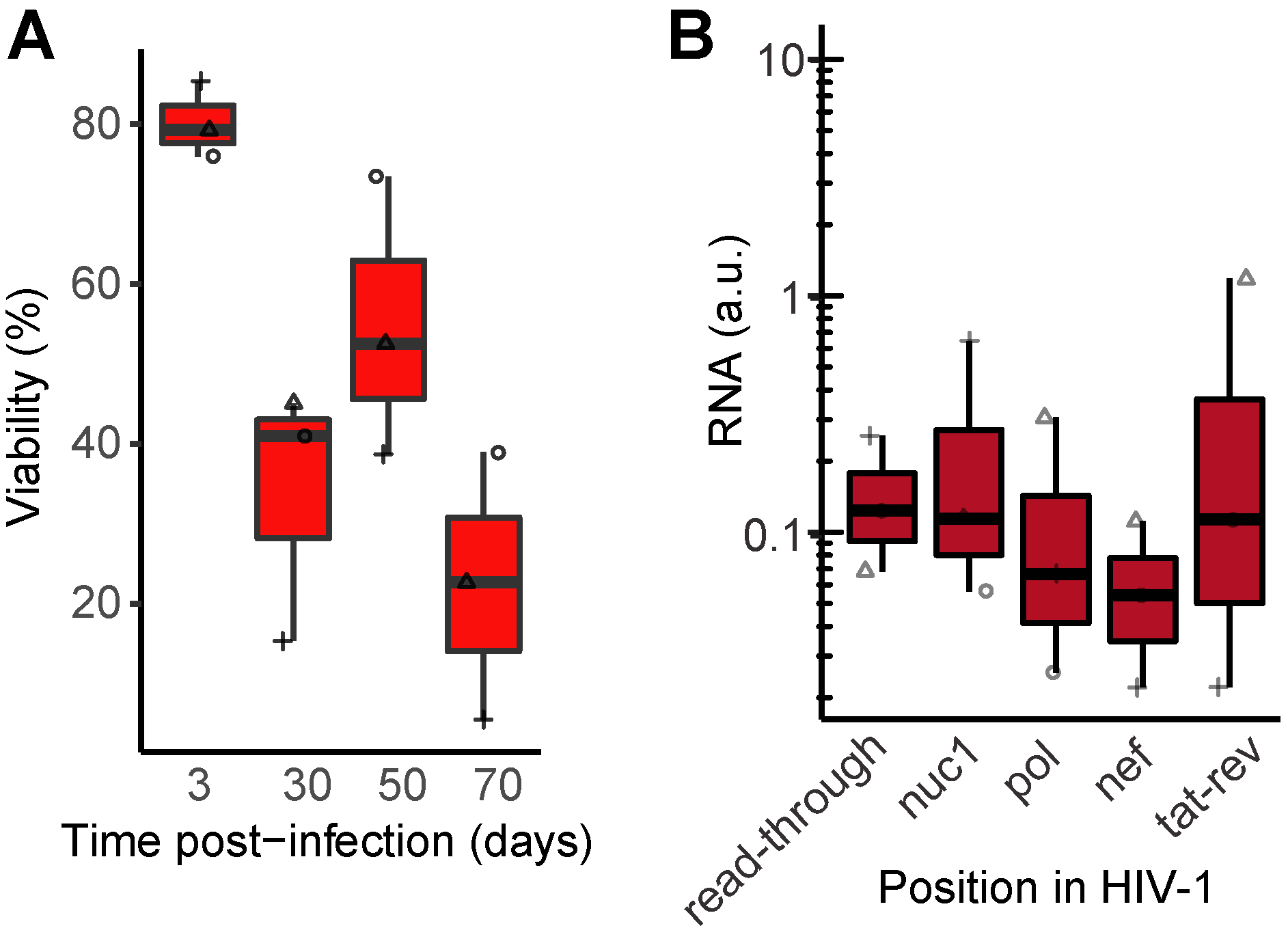

Supplement: S3 Fig — (A) Viability of Bcl2-cultures as measured by a membrane-permeable dye (n = 3). (B) CA-RNA levels originating from the provirus were quantified by RT-ddPCR in primary HIV-1 Bcl2 cells at 50 dpi. Probes were as in previous results (S1A Fig) (n = 2). (TIF) [file ppat.1008264.s003.tif]

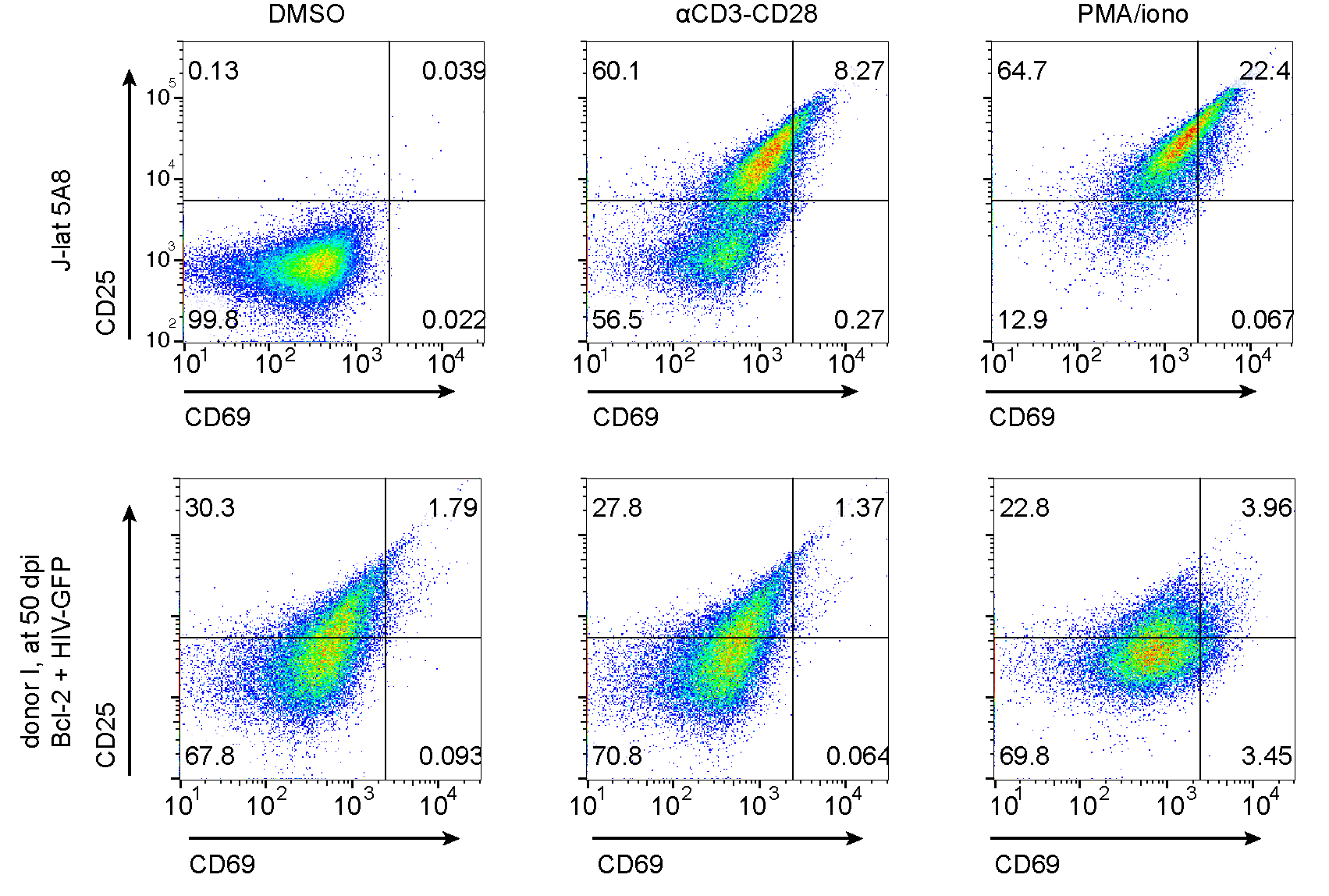

Supplement: S4 Fig — J-lat 5A8 cells (upper panels) and example of primary Bcl2 cells with HIV-1-GFP at 50 dpi (lower panels) were exposed to DMSO (left), antibodies against CD3 and CD28 (middle), or PMA/ionomycin (right) for 48 hours prior to flow cytometry analysis using labeled antibodies against surface markers CD25 and CD69. (TIF) [file ppat.1008264.s004.tif]

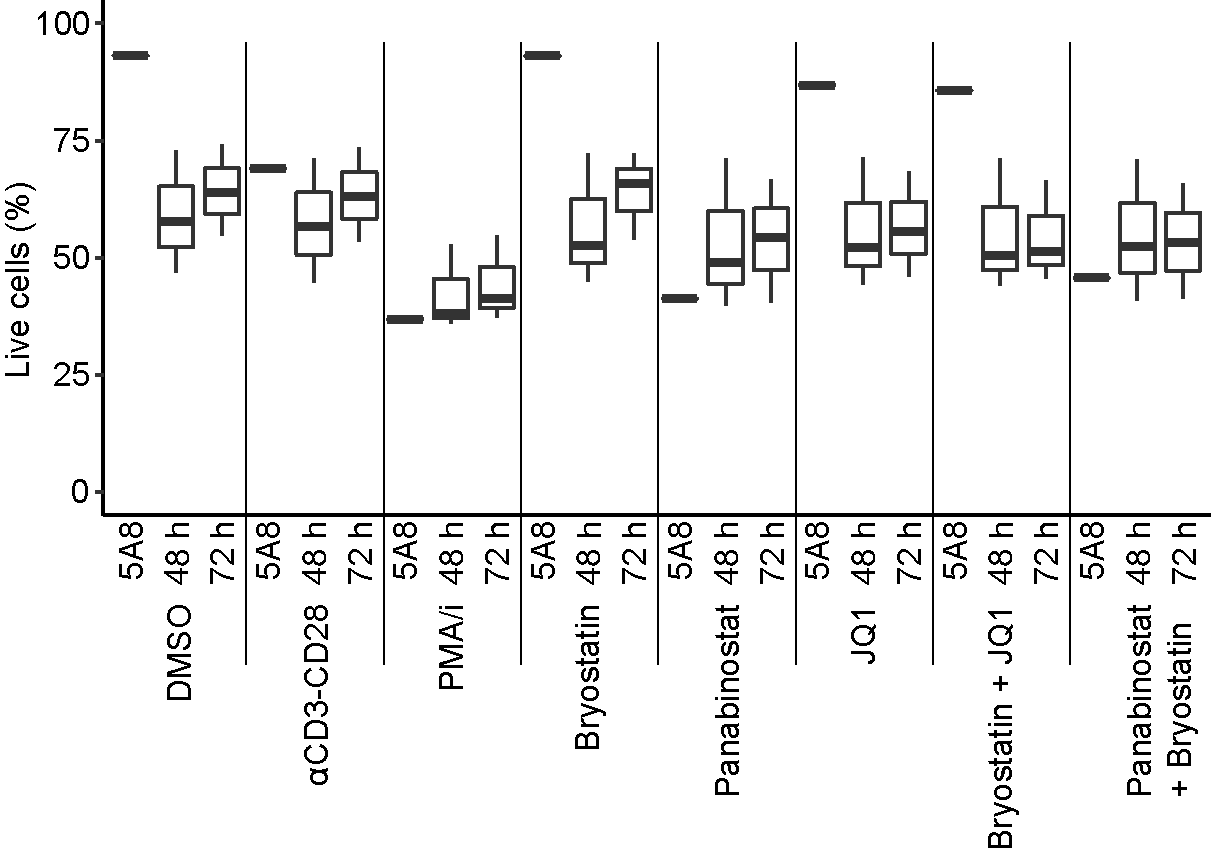

Supplement: S5 Fig — Boxplot showing the cell viability as determined by membrane integrity through LIVE/DEAD staining and flow cytometry. HIV-1 infected Bcl2 model cells from healthy donors (n = 3) were exposed to drugs for 48h and 72h. J-lat clone 5A8 was used as control. (TIF) [file ppat.1008264.s005.tif]

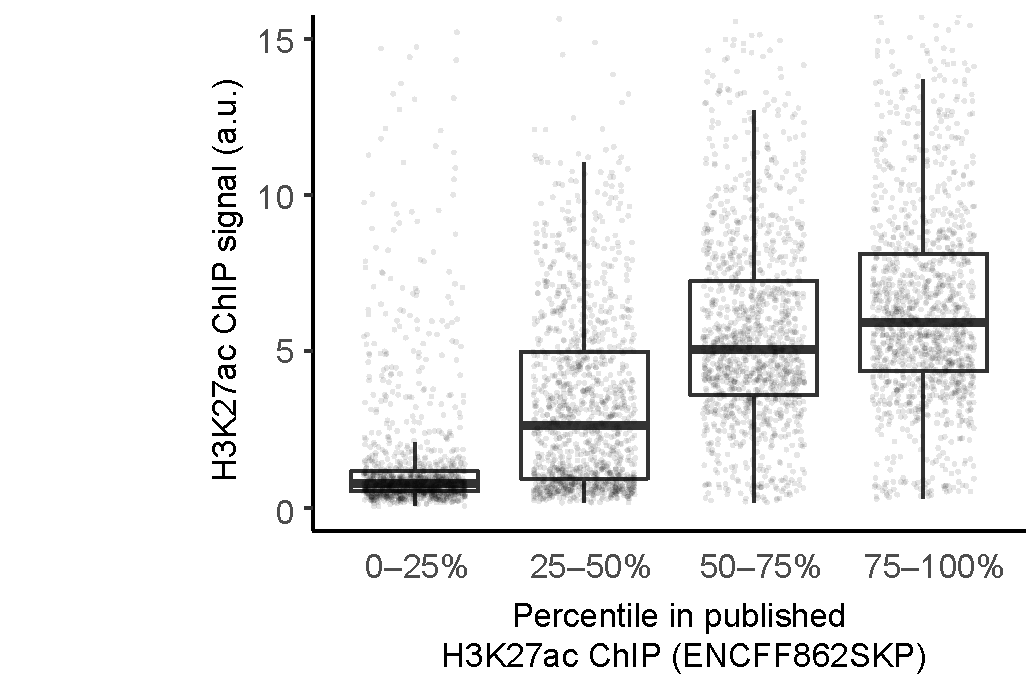

Supplement: S6 Fig — Boxplot showing the H3K27ac ChIP signals (resting CD4+ T-cells) calculated in 2kb-probes centered around the start of genes. Published ChIP data (ENCODE ENCFF862SKP) were processed in the same way and grouped in quartiles. All individual data points are shown. (TIF) [file ppat.1008264.s006.tif]

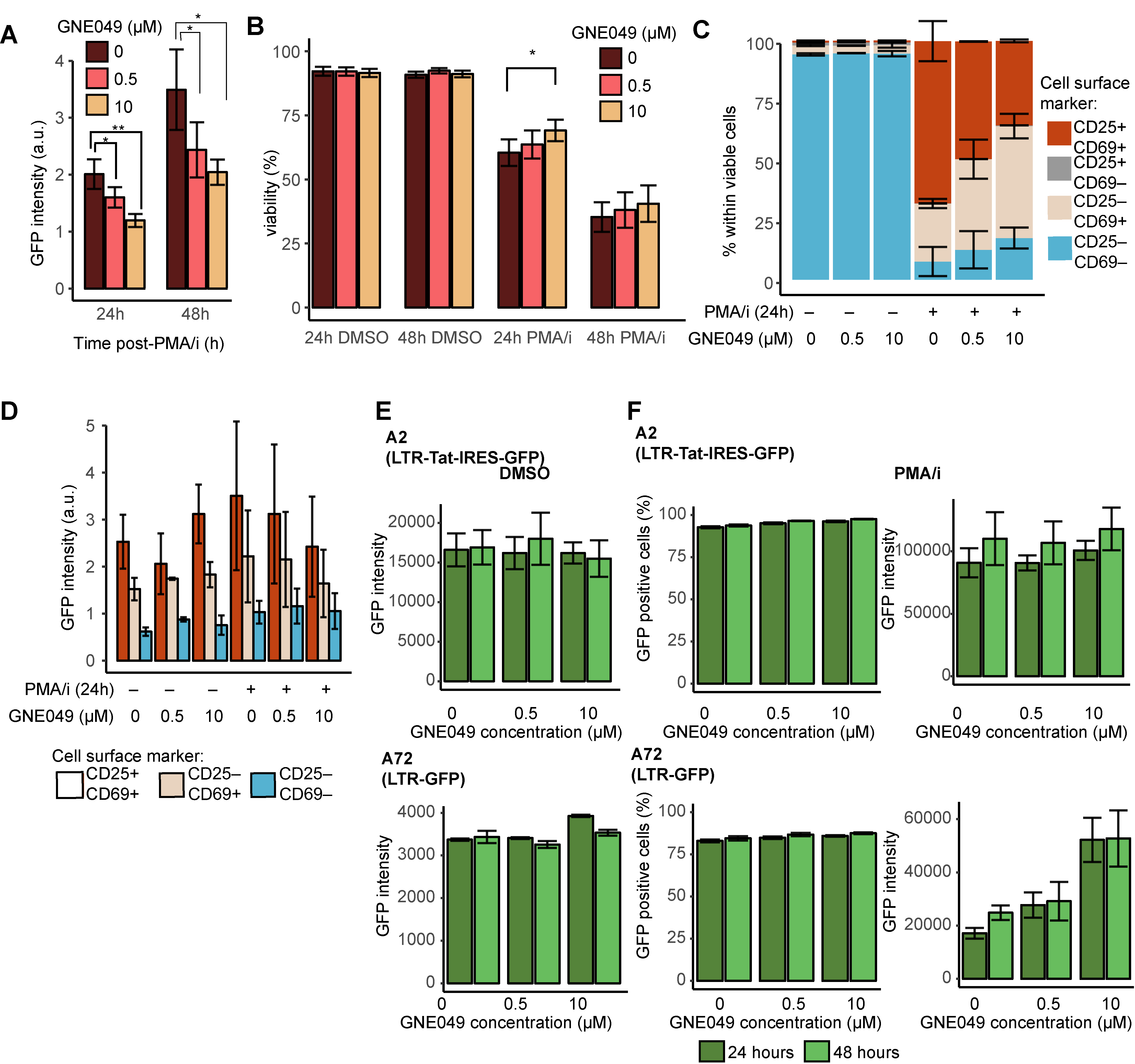

Supplement: S7 Fig — (A) GFP intensity in 5A8 GFP+ cells treated with GNE049. (B) Cell viability of activated 5A8 cells increase after treatment with GNE049. Cells were exposed to GNE049 or DMSO for 3 hours prior to treatment with PMA and ionomycin (PMA/i) or DMSO. After 24 or 48 hours, cells were stained with a LIVE/DEAD membrane-permeable dye and fixed; thereafter cells were analyzed by flow cytometry (n = 7). (C) The appearance of surface markers for activated cells (CD25 and CD69) after T-cell activation and GNE049 treatment (n = 2). (D) GFP intensity of cells in panel C. (E) In A2 and A72 cells, GFP intensity after GNE049 treatment (3h) followed by DMSO for 24 or 48h (F) In A2 and A72 cells, percentage of GFP positive cells and GFP intensity after GNE049 treatment (3h) and stimulation by PMA/i for 24 or 48h. *p<0.05, **p<0.01 paired t-test. (TIF) [file ppat.1008264.s007.tif]
