# Supplementary material for: Chromatin maturation of the HIV-1 provirus in primary resting CD4+ T cells
Source: PLoS Pathog. 2020 Jan 30;16(1):e1008264. doi: 10.1371/journal.ppat.1008264 (PMC6991963; doi:10.1371/journal.ppat.1008264)
Supplement: Attachment — Submitted filename: rebuttal_v2.docx [file ppat.1008264.s011.docx]

We are grateful for the insightful comments from the reviewers and editors. Based on the input we have substantially revised the manuscript to address all of the issues raised.

Major experimental changes to address the concerns of the model system and interpretations include:

1) We have increased the dataset with many more data points.
2) As we have more data points, we have been able to perform statistical analyses to a much higher extent.
3) We have included a third model system, primary T_EM_ cells HIV *in vitro* infection, where we have tested an expanded panel of LRAs.
4) The final and important enhancer section has been substantiated with several new experiments, including a new previously untested LRA targeting the enhancer functionality.

Along with the experimental improvements, we have also carefully revised the text.

The main conclusions remain from the earlier manuscript but are strengthened here. We believe that these changes have substantially improved the manuscript.

Below is a detailed point-to-point response.

**Part I – Summary:**

**Reviewer #1:** This manuscript deals with the important problem of HIV latency, the major barrier to curing the infection.  Using cells from patients and a primary cell model of latency, the authors have explored the mechanism by which transcriptional silencing of the provirus occurs in latently infected cells.  They find that repressive chromatin marks including H3K9me3 and H3K27me3 associated with the provirus.  However, some proviruses also show the enhancer mark H3K27ac, and it is those proviruses that can be induced following T cell activation.
This is an interesting and potentially important paper.  Because of the low frequency of latently infected cells in vivo, it is technically challenging to probe epigenetic modifications affecting HIV transcription is cells from patients.  This study makes an impressive effort to study epigenetic modifications in a primary cell model of latency.  The paper could be improved by considering the following points.

**Reviewer #2:** The authors develop a primary cell model, using some methods previously reported. In doing so they use a single laboratory strain of HIV, encoding 6 crippling mutations, and GFP in place of Env. Some cells are also transduced with Bcl2, although the authors claim without directly showing it, that Bcl2 trasnduction levels are so low that they believe that a minority of cells are producing "factors" due to the presence of the Bcl2 gene, and somehow supporting the other infected cells.

Of most concern, by Fig. 3 they show that their model cells grown for many days in culture lose the ability to respond to global activating signals (baseline GFP 1% increased to 1.3%) but "respond" (increase to 2%) when exposed to a combination of JQ1 and bryostatin that would be expected to be toxic to primary cells.

Given these caveats around the model system, and the lack of any validation in unmanipulated infected cells or other models, the conclusions drawn must be viewed with caution.

The Bcl2 construct is only present in a subset of the cells, as the 5´LTR primer/probe set perfectly identifying the Bcl2 promoter, is found in 1-4% of the parental cells before HIV-1 infection (Fig 2C), and maximally in 7% of the cells at 90 dpi (Fig 3C). The discussion on the Bcl2-containing cells acting as feeder cells producing factors to sustain growth has been moved from the result section to the discussion section as we have not tested this hypothesis. We now merely present it as a possible explanation.

It is correct that the model cells only modestly activate the provirus, but they do respond to global activation by anti-CD3-CD28 or PMA/i. This was phenotypically assessed by accelerated cell growth, pronounced cell lumpiness, rapid media turnover, and cell death (text p12, l276), although the cells largely failed to present the surface markers of T-cell activation (S4 Fig). We have quantitated the toxicity in the primary cells and show that the combination of bryostatin and JQ1 is among the middle-of-the-range among the different treatments (S5 Fig). Cell death on its own may be an inducer of the provirus, as we now discuss with reference Khan et al 2015. We agree with the reviewer that the response to LRAs is very low here. Indeed, we want to make the point that the latently infected cells remain refractory to proviral activation. We have clarified this in the text. As now noted in the introduction (p5, l 120), the effect of LRAs when tested in patients is lower than in most in vitro experimental systems.

As suggested, we have added unmanipulated infected cells, i.e. primary CD4 cells from HIV negative donors *in vitro* infected with the reporter HIV-1. These cells have a life-span of approximately 2 weeks. In these cells we have studied the unstimulated proviral transcription and the ability for T-cell activation or an expanded panel of latency reversal agents to activate the provirus (Fig 1C, D, F).

**Reviewer #3:** Review of PPATHOGENS-D-18-02102 manuscript: “Chromatin maturation of the HIV-1 provirus in primary resting CD4+ T cells by Lindqvist et al.

In this report, the authors attempt to characterize the epigenetic profile along the HIV-1 provirus in infected primary resting CD4+ T cells from the beginning of the viral infection up to the establishment of proviral latency. Despite the fact that this is an interesting issue to study in the HIV field and that the manuscript is clearly written, major problems have to be addressed. Indeed, the majority of the results presented by the authors could be differently interpreted. Moreover, the absence of statistical analyses, especially in the highly heterogeneous environment of primary cells studied here, is an additional major problem. The resolution of all the figures has to be drastically improved for a better understanding of the results presented in the manuscript. Therefore, the data presented here do not allow to convincingly establish the epigenetic landscape along the HIV-1 provirus in infected primary CD4+ cells.  

- Legends of all the figures related to Chromatin Immunoprecipitation assays require more information regarding the protocol used by the authors, regarding the manner the data are presented, normalized and interpreted, regarding the statistical analysis performed when present,… These improvements are required to understand the data.

- Fig. 1A : to better understand the relevance of the results presented by the authors, more information about the statistical analysis performed is mandatory. Indeed, despite the p-values, the reviewer is not convinced by the conclusion: “The abundance of mature multiply-spliced transcripts (tat-rev) was significantly lower (p<0.05) than the abundances of five unspliced products”. In addition, increasing the number of patients samples could strengthen the results. 

- Fig 2B : the reviewer does not agree with the conclusions drawn by the authors regarding the decline of HIV-1 infected cells since an increase was observed between 50 and 70dpi and between 90 and 120dpi, the last measurement. The authors cannot exclude that the number of HIV-1 infected cells could still increase after 120dpi. In addition, the lack of statistical analysis is again a major problem preventing a correct evaluation of the data. 

- Fig 2F : at 3dpi, a detection of all HIV-1 transcripts should be observed, maybe with an exception for tat-rev according to the data presented in Fig. 1A (but discussed above), in correlation with the GFP expression observed in Fig. 2E. However, no detection of these transcripts was observed. The authors should at least comment this issue.

- Figure 3C : (lanes 257 - 259) “T-cell activation by both αCD3-CD28 conjugation and PMA/ionomycin resulted in a small, but significant increase (p<0.05) in cells with activated provirus, compared to untreated cells, but only under certain conditions.” This conclusion is only correct for 50dpi and the authors should add more comments about the results observed at 70dpi, where no reactivation was observed. In addition, a constant decrease of GFP+ cells was observed following DMSO treatment but again, no explanation for this “strange” result was given by the authors.

- Fig 3D : lack of statistical analysis between some conditions : DMSO vs Panabinostat (putative induction of proviral activation) and DMSO vs Bryostatin + JQ1. In addition, the importance of a sequential treatment with different LRAs has previously been reported by others (Bouchat et al., EMBO Mol. Med., 2016) and such experiments are missing in the present manuscript. In addition, a combination with a DNA methyltransferase inhibitor could increase the level of reactivation in those HIV-1 infected primary cells and should be tested by the authors before performing the other assays in “activated conditions”.

- Figures 4A and 4B : in the “Results” section corresponding to these figures, no explanation was given regarding the choice of the nucleosomal localizations. Moreover, the quick conclusion (lanes 279 - 281) that is only correct for some points but totally wrong for the majority of the points : Fig. 4A nucleosome 6, Fig. 4B nucleosomes 1, 25 and 35 for which an increase was observed. In addition, the lack of statistical analysis prevents the interpretation of the data. 

- Lane 283 : “Active chromatin marks remain on the provirus” : the title of this section is partially true for the H3K27ac mark but not for the H3K4me3 mark that seems to disappear at 50 and 70dpi (Fig. 4C). The title should therefore be modified as follows: “Active chromatin marks along the provirus decrease over time following HIV-1 infection”

- Figure 5A : probably due to technical issues and/or to the heterogeneity of the primary cells, no key conclusion can be made about the increase of the negative epigenetic mark H3K9me3 that successively increases and decreases between 30 and 90dpi at nucleosomes 1, 25 and 35. More importantly, the lack of statistical analyses, associated with the important standard deviation observed, drastically decrease the strength of the results presented (same problems for Figure 5B).

- Figure 6A: the normalization of the results is difficult to understand without explanation. A representation of the epigenetic marks recruitment along the provirus in the two conditions (“mock-treated” and the treated one) should be presented by the authors and will give an easier message. In addition, no explanation is given regarding the levels of negative epigenetic marks that remained constant following TCR activation (whereas one would expect a decrease of those negative epigenetic marks following activation).

The data are rather heterogeneous as the reviewer points out. This is expected from the use of primary cells. To be able to strengthen the conclusions, we have expanded the data set for this revision. This has led us to be able to add statistical analyses wherever it is appropriate. We have also taken a more stringent approach to the interpretation. We apologize for the previous low resolution of the figures, they have now been uploaded in a different format to avoid quality loss.

Legends: We have expanded the legends to include more information on the ChIP assay, normalization, presentation, and the precise statistical analysis used in each panel. We have also included more details in the methods section.

Figure 1A: we have doubled the number of HIV-positive individuals (from 5 to 10) from whom we have obtained PBMCs. As the reviewer points out, the data varies strongly between the individuals as demonstrated by the visualizations, but consistent trends can be seen within samples. We have used the Wilcoxon signed rank test as we now describe in the legend. As the reviewer predicted, by inclusion of more individuals, the statistical significance between *tat-rev* and the rest of the primers were only maintained for 5’ primers *TAR* and *nuc-1*. This is changed in the text.

We have also expanded and substantiated this section to include resting CD4 T-cells (Fig 1B) as well as the CD4 depleted T cells (S1D Fig) and data from uninfected individuals (S1B Fig).

Figure 2B (Now Fig 3B): We agree that we misstated the data. There is no statistical significance to the potential decrease in HIV-1 frequency. We have now calculated at which point we can detect a statistically significant difference from the Bcl2-only cells instead. The point we want to make here is that during the first 90 days (when the data is collected) we are able to reliably detect the provirus in the cells. We have not gathered information on the days beyond 120 days as the viability of the cultures drastically decrease, however, in patients the HIV-1 infected cells are thought to be very long lived.

The text has been adjusted and statistical analyses have been added.

Figure 2F: Yes, given the GFP levels at 3 dpi we expected measureable RNA levels. The discrepancy between protein and RNA levels could be a result of lingering proteins. The RNA measurements of the Bcl2 model cells are rather noisy as the viability of the culture is rather low (approximately 50-70%) (see Fig S4, DMSO). In the data from flow cytometry or chromatin we specifically analyze living cells through live/dead discrimination or ficoll separation. We have expanded on this point in the text, notably p11 l 261 “This was surprising, as we could not detect significant RNA levels downstream of nuc1 (Fig 2F). This discrepancy might be due to technical issues or that transcription at 3 dpi is strongly reduced and the observed protein levels are lingering from earlier translation”. We have also included a section in the Discussion on the viability issue.

As for comparisons with Fig 1A, the inclusion of the new samples in Fig 1A (together with Fig 1B) now make the data consistent.

Figure 3C: The decline of GFP in resting cells we think comes from more cells transitioning from productive to latent infection and/or infected cells acquiring a “deeper” latency (i.e. less prone to reactivation), as heterochromatin data in Fig 5 (especially the compaction, panel C) show. This issue is elaborated on in the revised text.

A significant effect could be observed at 30 and 50dpi. We have changed the visualization of the data to better show this and given more details on the statistical testing. We have added “At 70 dpi, no effect could be determined, possibly as a consequence of few GFP-positive cells in the unperturbed state and poor cell viability.” to answer the concern of the 70dpi point.

Figure 3D: We have added the suggested drugs with the same concentrations and combinations as in Bouchat et al 2016 for the analysis in the primary T_EM_ cells. It is very possible the DNA methylation plays a role in the long-term latency. However, unfortunately, we did not have the capacity to redo the whole panel of Bcl2-cells to include the suggested drugs. Given the low dynamic range of the assay and the failure of almost all drugs to induce latency reversal in the Bcl2-system, we estimate that other assays will be better utilized to test the LRA activity of these drugs. These experiments are likely to yield interesting results but they are not within the scope of this study.

Figures 4A and 4B (now Fig 6A and 6B): We have now included justification of the four primers, basically they have been designed at the laboratories of C. Van Lint, E. Verdin and M. Ott. The conclusions from these data have been redrawn to be better justified. The inclusion of more data points in this expanded dataset have allowed us to perform comprehensive statistical analysis and thereby draw more precise conclusions. Statistical analysis is included here as elsewhere in the manuscript.

Lane 283 (now 337): The title is modified to “Active chromatin marks on the provirus”. We agree that this sentence in the previous manuscript misrepresented the data. We have now performed the statistical analysis on the much expanded dataset. This reveals that both chromatin marks are now present.

Figure 5A: We have performed the statistical analysis on the expanded dataset, and present the data in a more objective manner in the result section. Though the data is heterogeneous (especially at 50dpi and beyond), at several points can we detect statistical significance of heterochromatin accumulation as well as loss of accessibility.

Figure 6A: According to the reviewer’s suggestion to facilitate the interpretation, we now present the data in this panel (present 7A) with the data split in DMSO and PMA/i treated conditions, normalized to input.

The fact that the heterochromatin marks remain constant is discussed (p18, l440). We interpret the finding that heterochromatin marks at the provirus are not affected by T-cell activation as an indication that latency of these proviruses are not reversed, at least not to a level that we can reliably measure it in this experiment. This is consistent with heterochromatin being more refractory to activation, a key point of the study.

--------------------

**Part II – Major Issues:**

Reviewer #1: 1) Most of the work is done in a complex primary cell model.  Such models have provided many insights into HIV latency, allowing the discovery of many latency reversing agents.  However, it has proven much harder to reactivate latent HIV in vivo or ex vivo with patient cells with latency reversing agents, and clinical trials with agents that work well with primary cell models have been disappointing. This suggests potential differences between the model cells and the latently infected cells that persist in vivo.  This possibility should be acknowledged and discussed.

2) The authors show evidence of active transcription of the HIV provirus in latently infected cells but also present evidence for the sequestration of the proviruses in heterochromatin.   This seems to imply that the state of the provirus is different in different cells.  The authors should clarify the discussion of this point and ideally provide some more direct evidence on this point.

3) The key conclusion that the proviruses that are actively transcribed following T cell activation bear enhancer chromatin marks is based on the loss of this signal and a comparison with ChIP seq data from cellular genes.  The description of these key experiments could be improved and ideally supported by additional controls.

1) We agree completely with the reviewer about the disappointing results from clinical trials with LRAs and this point is brought up in both the introduction (p5, l118) and the discussion (p19, l460-481) sections.

2) To get direct evidence of the different states of the provirus in different cells we performed re-ChIP, where we sequentially ChIPped the same sample for two different antibodies (Fig 6F). This revealed that the enhancer chromatin appears at distinct proviruses from the heterochromatin. Further, we put the results in the context of the emerging field of phase-phase separation. Given that the heterochromatin is compacted (Fig 5C), it is most likely decorated by HP1 as observed previously (du Chene et al 2007). HP1 in turn promotes liquid droplets by phase separation within the nucleus (Larson et al 2017), which would exclude active marks such as H3K27ac in heterochromatin droplets (Gibson et al 2019). The HIV-1 provirus is speculated to be contained in a liquid droplet. We expanded on this in the discussion section (p18, l438).

3) The section with enhancer chromatin has been rewritten and expanded with several new experiments (H3K4me1 and H3K27ac ChIP, re-ChIP, CBP/P300 enhancer inhibition by GNE049, flow cytometry) presented in Fig 7C–G. We have also performed a simpler and more intuitive analysis of the genome-wide effects of T-cell activation on H3L27ac levels (Fig 7B).

Reviewer #2: 1) The relative abundance of transcripts is reported in Fig. 1 but lacks controls for efficiency of amplification of different products (see eg Yukl SA, STM 2018).

2) Perhaps I cold not see this due to quality of figure reproductions but no statististical analysis for any of the reported changes seems to appear in Fig 1-2, and 4-6

1) This has been added (Fig S1C). We now specify that we have only included data points where we also have amplification from the genomic DNA, except for *tat-rev* (splice product) and *polyA* (polyadenylation of mRNA) that have no DNA counterpart. We are using the same primers/probes as in Yukl et al 2018.

2) Statistical analyses together with descriptions have been added to all the panels and legends of all figures.

--------------------

**Part III – Minor Issues:**

Reviewer #1: 1) The Introduction could be rewritten to remove some slight inaccuracies and suboptimal citations.  In general, the authors should cite the first studies to prove a particular point and should make sure that they have precisely described the findings of the cited papers.  For example, the first sentences in the  Introduction seem to imply that HIV only integrates into cells that survive in the infection.  This may be the case for resting cells as described in one of the cited references, but in activated cells, cell death follows integration and viral gene expression.

2) For the experiment should in Fig. 1a, the authors should clarify whether they used purified resting CD4+ T cells.  If not, the results may reflect active transcription from infected cells that became activated in vivo.  Also, controls proving that the observed signals are coming only from viral RNA should be described

3) If Fig. 1d, it is unclear why uninfected cells give a signal with HIV probes.

4) The authors use indirect assays to prove that most of the infected cells in their model system carry intact proviruses.  Although tangential to the main points of the paper, this analysis could be substantiated with full genome sequencing.

1) We have rewritten the introduction to be more precise, better portray the current knowledge and improve the accuracy of the citations. The exemplified sentence has been removed.

2) In the initial manuscript, we had used PBMC. Here we have added more patients and also studied specifically resting CD4^+^ T cells (Fig 1B).

Also, we have now added controls for RNA isolated from 4 HIV-1 negative donors (Fig S1B), showing the background signal from the ddPCR reactions for each primer/probe set.

3) The *5´LTR* signal is present as the corresponding probes also bind to the Bcl2-promoter in Bcl2 cells. The *gag* and *env* signals represent the background noise in this particular experiment (0.1-0.3%). Note the log10 scale and that a single point for gag creates the extended box in panel 2C. We agree with the reviewer that this signal ideally should be lower. Also in the uninfected T_EM_ cells we actually detect a similar fraction of seemingly HIV-1 positive cells. However, for each experimental setup, we maintain a clear margin between infected cells and negative control cells. In the data from PLWH (Fig 1A–B) genomic DNA was isolated specifically. However, to spare precious sample, DNA from the HIV-1 infected Bcl2 cells was isolated from the input samples after chromatin isolation. We noted that the ddPCR from input chromatin was noisier that the ddPCR from specifically isolated genomic DNA. This discrepancy in DNA isolation is now described in the methods section.

4) We have improved the analysis on the intact proviruses with more data points and statistical analysis (Fig 2D,E). The full genome was sequenced as part of the genome-wide analysis (Fig 7B) where the entire provirus could be recovered. However, a proper answer to this question would require single cell (provirus) sequencing. Although potentially interesting, as the reviewer points out this is tangential to the main points and not within the scope of this study.

In the chromatin experiments we normalize to the H3 level, so that any deletions, mutations or lower signal due to APOBEC-induced point mutations will be considered.

Reviewer #2: Several introductory statements seemed overinterpreted or misinterpreted, and should be reformulated:

line 85 Reactivated latently infected T-cells have developed mechanisms that allow cell division without activating virally-induced cell death pathways.

cells in this study were isolated and analyzed after maximal stimulation. It is not clear that such cells would actually survive in vivo

Line 85: We have now carefully gone through the text and reformulated much of the introduction to avoid over interpretation or misrepresentation. We have carefully gone through all citations. The above sentence is removed.

Yes, it is true that the cells are maximally stimulated. We bring up the viability issue in the discussion section (p19, l468).
