# Supplementary material for: Chromatin maturation of the HIV-1 provirus in primary resting CD4+ T cells
Source: PLoS Pathog. 2020 Jan 30;16(1):e1008264. doi: 10.1371/journal.ppat.1008264 (PMC6991963; doi:10.1371/journal.ppat.1008264)
Supplement: Attachment — Submitted filename: rebuttal_rev2.docx [file ppat.1008264.s012.docx]

Point by point response to reviewers:

We are again grateful to the reviewers for providing constructive feed-back. In this revision, the experimental additions are:
1) we have included a new HIV-1 latency model system, A2 and A72 (Fig 7G, S7E,F Fig);
2) we have added ChIP (Fig 7C) and ddPCR (Fig 7F) data after GNE049 treatment to show the effect on chromatin marks H3K27ac, H3K4me1, and transcription;
3) we present the requested cellular viability measurements throughout the time course (S3A Fig).

We have also revised the text in several places as suggested by the reviewers specified below. Several new references are added to put our results in a wider perspective.

**Part I - Summary**

Reviewer #1: PLOS Pathogens
Chromatin maturation of the HIV-1 provirus in primary resting CD4+ T cells (Birgitta Lindqvist, Sara Svensson Akusjärvi, Anders Sönnerborg, Marios Dimitiou, J. Peter Svensson) (PPATHOGENS-D-19-01861)

The authors added comments, corrected or performed additional experiments to address the majority of the reviewers concerns. However, some points are still not convincing and need to be further addressed.

Reviewer #2: Overall, it seems to me that the authors have addressed most of the questions raised by reviewers, though some caveats in this study remain and could be discussed.

The major caveats of the primary cell latency model include a low basal level of GFP+ cells and low induction suggested by GFP+ cell rate, which was pointed out by reviewers in the major suggestions. These caveats are in the nature of the bcl-2 primary cell model. They were well addressed by the authors by revising the text and explained the data more extensively. Increasing the number of donors and substantial statistical analysis might also have helped to clarify the theory. However, it’s noteworthy that the caveats associated with the model do significantly weaken the conclusions. The initial frequency of infected cells was around 10% (line 218.) After weeks of culturing, GFP+ rates decreased to 1%, which was lower than 2% even upon stimulation. It suggests that 80-90% of HIV provirus were resistant to the reactivation, which should be kept in mind when interpreting the data in figures 5 and 6.

I doubt that switching between Jlat and bcl2 model (especially in figure 5-7) added any value to the study other than technical convenience. Epigenetic character is heavily system dependent. I don’t think that the data using primary cells and cell lines are comparable or interchangeable.

Also, I understand epigenetic is the main focus of this study. But, other cellular factors likely contribute to the transcriptional status of provirus together with epigenetic factors, which should at least be discussed.

Indeed, many of the latent cells are resistant to activation. We now state that more clearly and elaborated on this (lines 524–529). The fact that a large majority of intact proviruses are refractory to activation is an important issue as, if this translates to patients, this means that the reservoir as measured by intact proviruses is overestimated.

Switching between Jlat 5A8 and Bcl2 cells is purely for technical reasons, as stated by the reviewer. By including the 5A8 cells, we confirm previous data and as these experiments were done in parallel with the work on primary cells, we demonstrate technical soundness. However, it also brings some discrepancies, such as the differences in overall changes in H3K27ac levels after activation. As requested by reviewer 1, these discrepancies are further discussed (lines 538–540).

In the new revision we have expanded on the discussion on non-epigenetic factors that affect transcription (lines 522–524). Mainly, these references were previously only in the introduction section.

**Part II – Major Issues: Key Experiments Required for Acceptance**

Reviewer #1: Major comments:
1) In this work, the key conclusion that the proviruses actively transcribed following T-cell activation bear enhancer chromatin marks is based on the loss of this epigenetic mark and on a comparison with ChIP seq data from cellular genes. The reviewer 1 asked for additional experiments and controls to improve this conclusion. To address to this point, the authors used the GNE049 drug which depletes H3K27ac specifically at enhancers in 5A8 cells which have been described in Figure 8 to contain low level of H3K27ac before activation. The 5A8 cells are not the best model to conclude on the need to lose enhancer marks to reactivate HIV from latency since 5A8 cells contain high level of H3K27Ac in the provirus after PMA activation. It is therefore not surprising that increasing doses of GNE049 decreased the percentage of GFP intensity (Fig. 7D). To improve their experiments using the GNE049 drug, the authors should use a better HIV-1 latency model (in this case HIV-1 latently-infected cells with higher amount of H3K27ac enhancer mark) coupled to viral transcripts measurement.
2) No explanation is given by the authors regarding the levels of negative epigenetic marks that remained constant following TCR activation (Figure 7A). The authors responded by stating : “that heterochromatin marks at the provirus are not affected by T-cell activation as an indication that latency of these proviruses are not reversed, at least not to a level that we can reliably measure it in this experiment”. The authors should further discussed (in regard to previous work in the literature) the epigenetic marks H3K9me3 and H3K27me3 which remained constant after TCR activation and provide the percentage of GFP positive cells in absence and presence of TCR activation, supporting the absence of HIV-1 latency reversal.

1) To address the reviewer’s concern, we have added another model for HIV-1 latency reversal. Whereas the 5A8 J-lat cells contain the entire provirus, where factors unrelated to enhancer functionality may interfere with our interpretations, the combination of A2 and A72 cells is much more simplistic. These cells contain the HIV-1 LTR promoter and a short open reading frame with either Tat-IRES-GFP (A2) or GFP only (A72).

In the A2/A72 cell lines, the GNE049 effect on latency reversal was much stronger than observed in the 5A8 cells (Fig 7G, S7E,F Fig). This finding clearly shows that the enhancer functionality promotes latency of the HIV-1 promoter.

While performing these experiments, it was revealed that the low GFP amplitude after GNE049 exposure could be attributed to Tat. Possibly, this is a consequence of Tat acetylation that has been described previously (references included in manuscript). Even though the chromatin target of GNE049 is specific to H3K27ac at enhancers, it may also block Tat acetylation. This is however not the focus of the study presented here. As a consequence, the change of GFP amplitude (previously in Fig 7D,F) has been moved to the supplementary information (S7A–D Fig).

As for the levels of H3K27ac in unstimulated 5A8 cells, we have measured them to 0.9–1.5 (relative to H3, Fig 6D), compared to 0.8–0.9 as median in the donor cells. To address the reviewer’s concern that substantial H3K27ac levels are required, we performed H3K27ac and H3K4me1 ChIP 5A8 cells after GNE049 (or DMSO) exposure (Fig 7C). The results show that the drug acts on the proviral chromatin, and specifically decreases the level of H3K27ac (p<0.05 or p<0.001 depending on position).

As reviewer 1 suggests, we have also determined the effect of GNE049 on viral transcripts (Fig 7F). Somewhat disappointingly, we note only a slight but statistically significant (p<0.05) increase on the TAR region. All positions probing the spliced transcripts showed a minor increase in transcription after GNE049 treatment.

We believe these new results have substantially improved the study and we are grateful for the meticulous examination by the reviewer.

No explanation is given by the authors regarding the levels of negative epigenetic marks that remained constant following TCR activation (Figure 7A). The authors responded by stating : “that heterochromatin marks at the provirus are not affected by T-cell activation as an indication that latency of these proviruses are not reversed, at least not to a level that we can reliably measure it in this experiment”. The authors should further discussed (in regard to previous work in the literature) the epigenetic marks H3K9me3 and H3K27me3 which remained constant after TCR activation and provide the percentage of GFP positive cells in absence and presence of TCR activation, supporting the absence of HIV-1 latency reversal.

2) In the revised manuscript we have expanded on the discussion of H3K9me3 and H3K27me3 with references to the literature. We now also state the percentage of GFP positive cells in the absence and presence of TCR activation (lines 319–321).

We have expanded on the discussion of silencing epigenetic marks H3K9me3 and H3K27me3 and more carefully position our data in relation to previous studies by others.

Reviewer #2: no

**Part III – Minor Issues: Editorial and Data Presentation Modifications**
Please use this section for editorial suggestions as well as relatively minor modifications of existing data that would enhance clarity.

Reviewer #1: Minor comments:
1) Reviewer 1 mentioned that in Fig 2F: at 3dpi, a detection of all HIV-1 transcripts should be observed. However, no detection of these transcripts was observed. In Figure 2F, all HIV-1 transcripts were not observed with no detection of transcripts downstream nuc1. The authors responded to this point by stating :” This discrepancy might be due to technical issues or that transcription at 3 dpi is strongly reduced and the observed protein levels are lingering from earlier translation”. They also included a section in the Discussion on the cellular viability issue. The authors should provide information and/or additional figure regarding the cell viability. Same remark for Figure 3C at 70 days post-infection
2) Reviewer 3 observed variations throughout the proviral genome without statistical analysis of the significance of these variations over the time post-infection (Figure 5A). The authors answered by performing statistical analysis on the dataset. However, they observed statistical significance of heterochromatin accumulation at several points. Thus, the authors should be careful when stating (line 314): “The constitutive H3K9me3-mark appeared to be uniformly distributed over the proviral body by 30 dpi” and should further discuss these variations.
3) The authors should explain and discuss the contradictory results regarding the H3K27ac mark obtained in 5A8 and HIV-1 BCL2 cells (Figures 6E and 7A).

1) Cell viability measurements of the Bcl2 cultures throughout the time course are now included (S3A Fig). As for the comment on transcription at 3dpi, this was added in the first revision to as to explain the observed lack of transcription. We agree with the reviewer that it is speculative and have therefore removed it. The value of Fig 2F is that despite having low technical resolution we still observe a biologically and statistically significant transcription at the nuc-1 region. The transcripts of downstream regions are expected to be lower and the reasons for them not being observed here needs further experiments to be resolved (not within the scope of this revision).
2) We have revised this statement by removing “uniformly” as we, as the reviewer states, have weak evidence of this. The current statement better reflect the data. We have also added “However, the large variation between donors should be noted.” to highlight this fact (line 338).
3) We have expanded in the discussion on the contradictory results of the H3K27ac levels in the 5A8 cell line and the primary cells (lines 538–540).

   Reviewer #2: please see above
